# A Human Activity Recognition Algorithm Based on Stacking Denoising Autoencoder and LightGBM

**DOI:** 10.3390/s19040947

**Published:** 2019-02-23

**Authors:** Xile Gao, Haiyong Luo, Qu Wang, Fang Zhao, Langlang Ye, Yuexia Zhang

**Affiliations:** 1 Beijing Key Laboratory of Mobile Computing and Pervasive Device, Institute of Computing Technology Chinese Academy of Sciences, Beijing 100190, China; gaoxile17g@ict.ac.cn (X.G); yelanglang@ict.ac.cn (L.Y.); 2 School of Information and Communication Engineering, Beijing University of Posts and Telecommunication, Beijing 100876, China; wangqu@ict.ac.cn; 3 School of Software Engineering, Beijing University of Posts and Telecommunication, Beijing 100876, China; zfsse@bupt.edu.cn; 4 School of Information and Communication Engineering, Beijing Information Science and Technology University, Beijing 100876, China; zyx@bupt.edu.cn

**Keywords:** human activity recognition, indoor positioning, deep learning, Stacking Denoising Autoencoder, LightGBM

## Abstract

Recently, the demand for human activity recognition has become more and more urgent. It is widely used in indoor positioning, medical monitoring, safe driving, etc. Existing activity recognition approaches require either the location information of the sensors or the specific domain knowledge, which are expensive, intrusive, and inconvenient for pervasive implementation. In this paper, a human activity recognition algorithm based on SDAE (Stacking Denoising Autoencoder) and LightGBM (LGB) is proposed. The SDAE is adopted to sanitize the noise in raw sensor data and extract the most effective characteristic expression with unsupervised learning. The LGB reveals the inherent feature dependencies among categories for accurate human activity recognition. Extensive experiments are conducted on four datasets of distinct sensor combinations collected by different devices in three typical application scenarios, which are human moving modes, current static, and dynamic behaviors of users. The experimental results demonstrate that our proposed algorithm achieves an average accuracy of 95.99%, outperforming other comparative algorithms using XGBoost, CNN (Convolutional Neural Network), CNN + Statistical features, or single SDAE.

## 1. Introduction

With the development of the healthy life and smart home concept, human activity recognition (HAR) has been increasingly studied and applied in Human–Computer Interaction (HCI), and Mobile and Pervasive Computing [1]. One of the purposes of HAR is indoor positioning [2]. As the landmark of indoor positioning, elevators and escalators detect whether a human is currently taking them by judging moving modes to calibrate the indoor positioning results. Human physical motion recognition can also be used in indoor navigation by combining with the wireless signals [3]. Another feasible objective of HAR is static behavior recognition for safe driving [4], and scientific exercise [5]. Moreover, HAR can also be used for dynamic behavior recognition in healthcare monitoring [6]. This process will detect whether the patients or the elderly experience a sudden fall and raise the alarm promptly to protect the personal safety of the users. In addition, other applications include bilateral links for advertising, entertainment, games, and multimedia visualization guidance [7,8]. 

At present, the HAR methods are mainly divided into HAR based on vision [9,10] and HAR based on sensors. Vision-based HAR has high recognition accuracy, but it brings with it high power consumption and privacy problems. As for HAR based on sensors, the more sensors a user carries, the more detailed the classification items that can be achieved [11,12]. However, smartphones have inherent advantages (such as having various integrated sensors and computing ability, as an essential gadget in a human’s daily life), thus, resulting in smartphones becoming a prominent tool for HAR [13]. Generally, sensors-based HAR is performed in four fundamental steps: data collection, data segmentation, feature extraction, and classification.

As for data collection, the first part of HAR, there are multiple factors. The first is the pose of smartphones which may be typing [14], swinging [14], phoning [14], in pocket [14,15], in hand [15], and on waist [16]. The second factor is the collection frequency. The researchers have tried multiple possibilities such as 50 Hz [17], 76.25 Hz [18], 100 Hz [19], and 120 Hz [20]. Besides, there are various publicly available datasets that provide strong support for human activities recognition such as UCI [16], WISDM [21], HASC [22], and RealWorld HAR [23]. 

Following data collection is data segmentation. The existing works considered time segments of size 200 in 10 s [21], 300 in 7.5 s [5], 512 in 6.7 s [18], 50 in 1 s, and 128 in 2.56 s [13]. The authors of [14,24] have done research on the impact of sliding window length in indoor human motion modes and pose pattern recognition based on smartphone sensors and reveal that a window length between 2.5 s and 3.5 s provides an optimal tradeoff between recognition performance and speed for motion mode recognition.

According to the feature construct method, the current feature extraction methods of HAR are divided into the artificial feature construction method [15,20,21,25,26] and deep learning feature construction method [13,17,27,28,29]. Tran et al. [26] added the features of the frequency domain into consideration and Sang et al. [20] imported the fractal dimension. Khalifa et al. [30] introduced the concept of kinetic energy collection. Cao et al. [31] proposed a Group-based feature extraction method. However, to find the most efficient and effective features, the programmers must have prior expert knowledge, or they must do a large amount of empirical study to learn which features are useful [32]. To overcome the above limitation, many researchers have used a deep learning method for HAR, such as CNN (convolutional neural network) [33,34,35], a deep neural network [36], or a recurrent neural network [19]. Among them, many kinds of researches based on CNN have achieved remarkable results. Chen et al. [27] realized the HAR based on CNN and analyzed the influence of different parameters on classification accuracy. Ignatov et al. [13] combines the features extracted by CNN with the statistical features and introduced them into the classification network together to improve the accuracy of classification.

After extracting the feature of the sensor data, various classification methods have been tried for HAR. As traditional machine learning methods, Support Vector Machine (SVM) [25,26], Random Forest (RF) [37], Logistic Regression (LR) [20], eXtreme Gradient Boost (XGB) [20], and Light Gradient Boosting Machine (LGB) have been deeply studied. Sang et al. [20] compared the classification accuracy of different classifiers with the same features, such as LR, Decision Tree (DT), SVM, and XGB. Bayat et al. came from the perspective of classifier fusion and found that Multilayer Perceptron (MP) + LigitBoost + SVM will achieve the best classification effect. As for deep learning method, the classification network varies with the specific algorithm. Chen et al. used an elementary Convolutional Neural Network [27]. Almaslukh et al. put both the features of statistics and the features learned from the convolutional layers into the fully connected layer for classification [17]. The variants of the CNN are also commonly used for human activity recognition method [28,29]. Inoue et al. [19] utilized a deep recurrent neural network to realize activity recognition with high throughput which refers to the short time at a time from raw accelerometer data. After adding a fully connected layer behind the output of encoding layers, a single SDAE network can also be constructed for human activity recognition.

Table 1 lists the mainly HAR algorithms based on the different combinations of feature extraction methods and feature classification methods. As can be seen, the algorithms based on deep learning feature extraction methods often achieve better performance. 

Although many works focus on HAR, there are still many deficiencies in the accuracy, latency, and power consumption. The observation noise of the sensor is the key reason for the low recognition accuracy. Recently, stacked autoencoder (SAE), as a classical unsupervised learning algorithm, has shown high feature extraction [40,41] and data compression [42,43] performance that matches the current state-of-the-art [41]. Vincent et al. [44,45] modified the traditional SAE to learn useful features from corrupted data and developed the stacked denoising autoencoder (SDAE) that eliminates sensor observation noise by signal reconstruction. The SDAE model has the potential to eliminate noise and extract robust unsupervised feature in practice. Nevertheless, few researchers have used SDAE as an independent feature extraction module in HAR. A deep convolutional autoencoder (CAE) network proposed in [46] utilizes autoencoder to initialize the weights of the following convolutional layers. Another network named AE-LRCN [47] uses the autoencoder layer to remove the inherent noise of the input data. Thus, it is necessary to carry out the task of HAR based on features extracted from SDAE.

Unlike traditional HAR algorithms, this paper proposes an fusion method of Stacked Denoising Autoencoder [45] and LightGBM [48] for human activity recognition based on inertial sensor data of smartphone and highlights the classification of four different daily activities under three typical scenarios of human moving modes, current static behavior, and current dynamic behavior. The main contributions of this paper are as follows:

- We proposed a method which combines the feature extraction ability of deep learning with the classification ability of decision tree. We make advantage of SDAE to filter the occasional sensor noise (caused by the low-cost MEMS and complex human activities) and use the automatically obtained features for accurate human activity recognition The Boosting K-Fold LGB is used to realize accurate classification of the user behaviors.
- We proposed a little trick of k-Fold based on the idea of Boosting. By repeating the error classification samples in the validation set of the previous fold, the attention of the $n^{th}$ fold error samples can be improved in the $n+1^{th}$ fold training.
- We selected four datasets under three typical application scenarios, to verify the algorithm proposed in this paper and prove that this model can achieve high accuracy in multiple data sets and multiple classification problems.
- We also implemented the state-of-art algorithm based on XGB [49], CNN [50], CNN + statistical features [13], and single SDAE [2]. Then, we compared the proposed algorithm and the state-of-art algorithm on the same datasets.

The remainder of this paper is structured as follows: our proposed methodology is presented in Section 2. Section 3 describes and discusses our experiments. The conclusion is provided in Section 4.

## 2. Materials and Methods

### 2.1. System Architecture

**Figure 1:**
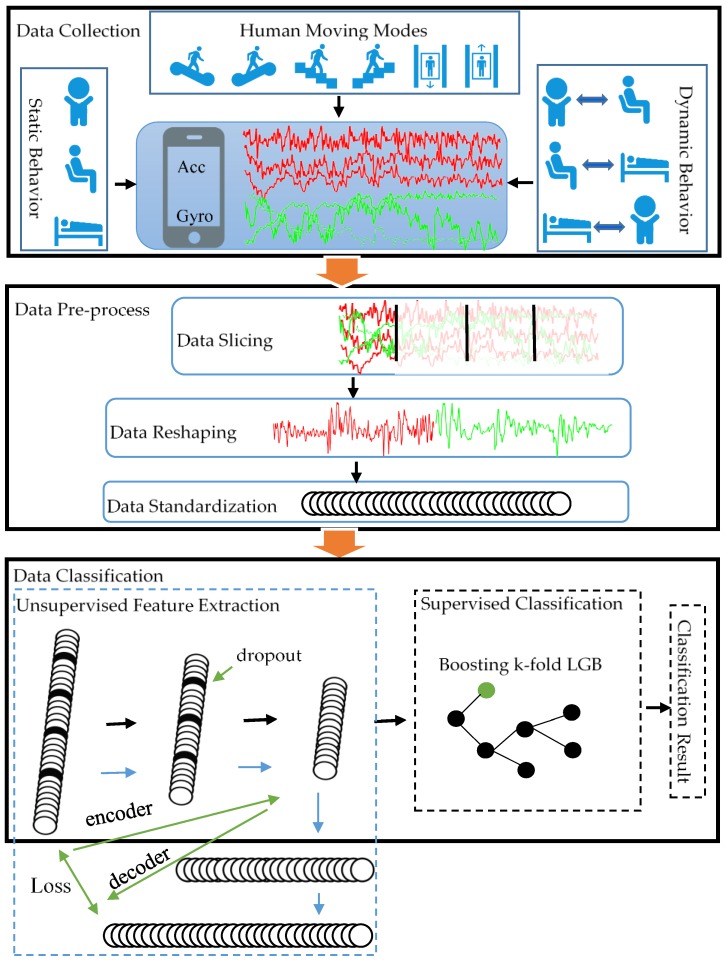
The architecture of the classification network. The solid black line in the data classification module represents the process of data classification while the solid blue line represents the encoding and decoding process of SDAE.

In this paper, we employ smartphone embedded sensors for human moving modes, static and dynamic behavior recognition. As shown in Figure 1, the proposed algorithm includes four steps including data acquisition, data preprocessing, unsupervised feature extraction based on SDAE, and supervised behavior classification based on K-Fold LGB.

For describing conveniently, we introduce the notations used in this paper. We use ℕ for the number of the sensors. Let ${Χ}^{k},k=1,2\ldots \mathbb{N}$ represent the original data collected from the $k^{th}$ sensor and $d^{k}$ represent the dimension of the $k^{th}$ sensor. After the sliding window with a size of w and a stride length of s, ${Χ}^{k}$ is divided into N samples, each of which is represented by $x_{i}^{k},i=1,2,\ldots ,N,x_{i}^{k}\in {\mathbb{R}}^{w\times d^{k}}$. After splicing and standardization, the input of the $k^{th}$ SDAE model will be obtained, which is represented by ${\vec{x}}_{i}^{k},{\vec{x}}_{i}^{k}\in {\mathbb{R}}^{wd^{k}}$.

### 2.2. Data Pre-Process

The data pre-process aims to change the sensor data collected at a fixed frequency into the input of the SDAE network. The specific processing process is described below.

**Data Segmentation.** In each experiment conducted by each person, the result is a sensor data sequence which has indefinite length. A sliding window with a size of 2.56 s is used to capture sample data on the different datasets. The final shape of the samples is shown in Section 3.1. 

**Data Reshaping.** To match the input shape of SDAE, it is necessary to reshape the sample obtained in the previous step. In this paper, we use the axis as the module to splice. If the sample shape after the first step is $\left(N^{\prime },P^{\prime },M^{\prime }\right)$, then the reshaped sample shape is $\left(N^{\prime },P^{\prime }\times M^{\prime }\right)$.

**Data Standardization.** This paper adopted the max–min standardization method before feeding the sample data into the SDAE network. The paper adopted the max–min standardization method. The following procedure is performed for the $i^{th}$ column of data $\left(x\left[:,i\right],i=1,2,\cdots P^{\prime }\times M^{\prime }\right)$ for the samples obtained from the previous step:

(1) $$x[:,i]=\frac{x[j,i]-\min(x[:,i])}{\max(x[:,i])-\min(x[:,i])},i=1,2,\cdots ,P^{\prime }\times M^{\prime },j=1,2,\cdots N^{\prime }$$

**Algorithm 1**. Data Pre-process**Input:** A time-series matrix d, sliding window size l, sliding window step s**Output:** A matrix of final train samples, A matrix of final test samples1. result = []2. Initialize the flag number start = 03. **while** start < len(d)4.  **if** start + l < len(d)5.   temp = d.iloc[start:start+l, :]            //data segmentation6.   reshaped_temp = reshape(temp, [1, l×d.shape [1]]) //data reshaping7.   result.append(reshaped_temp)8.    start = start +s9.  **end if**10. **end while**11. X_train, X_test = train_test_split(test_size = 0.3)12. //data standardization13. **for** col in X_train.cols14.  col_max = max(X_train.iloc[:, col])15.  col_min = min(X_train.iloc[:, col])16.  X_train.iloc[:, col] = (X_train.iloc[:, col] – col_min)/(col_max – col_min)17.  X_test.iloc[:, col] = (X_test.iloc[:, col] – col_min)/(col_max – col_min)18. **end for**19. return X_train, X_test

The pseudo code of Algorithm 1 shows the data pre-process implementation. This algorithm receives a time-series matrix $d=\left[d_{1},\cdots ,d_{i},\cdots d_{n}\right]$, a variable l represents the size of the sliding window, and a variable s represents the step of the sliding window. For matrix d, $d_{i}$ is a m-dimension vector where m represents the number of the axes of all the sensors. The algorithm output is defined by a2-dimension list named “finalsamples” with a shape of [n|s−1,l,m].

### 2.3. Unsupervised Feature Extraction

In this section, we introduce unsupervised feature extraction based on SDAE and demonstrates the effectiveness of the extracted features.

#### 2.3.1. Feature Extraction

SDAE come from a deep network scheme which stacks multiple denoising autoencoder together to learn complicated features [45]. Each denoising autoencoder consists of four layers: input, imnoise, hidden, and output layers. The hidden layer and the output layer are called the encoding layer and the decoding layer, respectively. To thoroughly learn the data variation rules of each sensor, we specifically construct a separated SDAE network. Each sensor data is passed into an SDAE network separately for data forward propagation and parameter reverse learning.

Assuming that there are $n^{k}$ layers of $k^{th}$ SDAE, then at the $l^{th}$ layer, a complete set of encoding–decoding operations is performed. Given the pre-processed vector of the $k^{th}$ sensor named $i_{{\vec{x}}_{i}^{k}}^{l}$, the imnoise layer of denoising autoencoder first transforms it by

(2) $$n_{{\vec{x}}_{i}^{k}}^{l}=f_{noi}\left(i_{{\vec{x}}_{i}^{k}}^{l};\theta_{noi}^{k,l}\right)$$

where $f_{noi}(\cdot )$ is the noising function, $\theta_{noi}^{k,l}$ is the probability of dropout in this paper. By using the dropout layer, a certain number of input sensor data are randomly chosen and forced to be zero. The encoding layers are trained to fill in these blanks and reconstruct these corrupted inputs of sensor data. Let $e_{{\vec{x}}_{i}^{k}}^{l}$ be the output of encoding layer, calculated by a function of

(3) $$e_{{\vec{x}}_{i}^{k}}^{l}=f_{enc}(n_{{\vec{x}}_{i}^{k}};\theta_{enc}^{k,l})$$

where $f_{enc}(\cdot )$ represents the encoding function and $\theta_{enc}^{k,l}$ is the noised-to-hidden parameters. The $e_{{\vec{x}}_{i}^{k}}^{l}$ obtained by the encoding function is the feature learned in the current layer. Let $f_{dec}$ represent the decoding function, $\theta_{dec}^{k,l}$ represent the decoding parameters, and the final output of the denoising autoencoder data will be expressed as

(4) $$d_{{\vec{x}}_{i}^{k}}^{l}=f_{dec}(e_{{\vec{x}}_{i}^{k}}^{l};\theta_{dec}^{k,l})=f_{dec}(f_{enc}(f_{noi}\left(i_{{\vec{x}}_{i}^{k}}^{l};\theta_{noi}^{k,l}\right);\theta_{enc}^{k,l});\theta_{dec}^{k,l})$$

In Formula 4, $\theta_{noi}^{k}$ is a super parameter that needs to be defined manually. While $\theta_{enc}^{k},\theta_{dec}^{k}$ are parameters that need to be trained and adjusted through the back-propagation process, where the loss function is defined to minimize the mean square error between the decoded data and the input data, which is

(5) $$(\theta_{enc}^{k,l}{}^{*},\theta_{dec}^{k,l}{}^{*})=\arg\underset{\theta_{enc}^{k,l},\theta_{dec}^{k,l}}{\min}{\left\|d_{{\vec{x}}_{i}^{k}}^{l}-i_{{\vec{x}}_{i}^{k}}^{l}\right\|}^{2}$$

In this so-called “denoising” way, we can reduce the influence from the inherent noise of sensor data collected by smartphones and focus on retrieving the information we need, or the so-called “useful features”. Then in the stacked structure, once the $l^{th}$ layer are trained, the SDAE scheme then leverages the outputs to train the $l+1^{th}$ layer. After fine-tuning of the layers, we obtain the final “useful features” by

(6) $$e_{{\vec{x}}_{i}^{k}}=f_{enc}(\cdots f_{enc}({\vec{x}}_{i}^{k};\theta_{enc}^{k,1});\cdots \theta_{enc}^{k,n^{k}})$$

When all the feature extraction tasks are completed, the features of each sensor are pieced together again to form the final feature vector $e_{{\vec{x}}_{i}}=e_{{\vec{x}}_{i}^{1}}|e_{{\vec{x}}_{i}^{2}}|\cdots |e_{{\vec{x}}_{i}^{\mathbb{N}}}$. Together with the label $y_{i},i=1,2,\ldots N$, it forms the input of supervised feature classification layer.

#### 2.3.2. Analysis of the Feature Performance

To evaluate the influence of the features extracted by SDAE, we leverage the inner-class dispersion matrix and the outer-class dispersion to describe the distribution of samples.

The inner-class dispersion matrix of the class $\Omega_{i}$ is defined as

(7) $$S_{W}^{\left(i\right)}=\frac{1}{N_{i}}\sum_{k=1}^{N_{i}}\left(X_{k}^{\left(i\right)}-m^{\left(i\right)}\right){\left(X_{k}^{\left(i\right)}-m^{\left(i\right)}\right)}^{T}$$

where the $X^{\left(i\right)}$ represents the $i^{th}$ class sample set and the $m^{\left(i\right)}$ is the mean of all the samples in $X^{\left(i\right)}$. The total inner-class dispersion matrix is defined as

(8) $$S_{W}=\sum_{i=1}^{M}P(\Omega_{i})S_{W}^{\left(i\right)}=\sum_{i=1}^{M}P(\Omega_{i})\frac{1}{N_{i}}\sum_{k=1}^{N_{i}}\left(X_{k}^{\left(i\right)}-m^{\left(i\right)}\right){\left(X_{k}^{\left(i\right)}-m^{\left(i\right)}\right)}^{T}$$

where M donates the number of the sample classes, and the $P(\Omega_{i})$ is the probability of the $i^{th}$ class samples in the total number of samples. For the outer-class dispersion, the dispersion matrix is defined as

(9) $$S_{B}^{\left(ij\right)}=(m^{\left(i\right)}-m^{\left(j\right)}){\left(m^{\left(i\right)}-m^{\left(j\right)}\right)}^{T}$$

(10) $$S_{B}=\frac{1}{2}\sum_{i=1}^{M}P(\Omega_{i})\sum_{j=1}^{M}P(\Omega_{j})S_{B}^{\left(ij\right)}=\frac{1}{2}\sum_{i=1}^{M}P(\Omega_{i})\sum_{j=1}^{M}P(\Omega_{j})(m^{\left(i\right)}-m^{\left(j\right)}){\left(m^{\left(i\right)}-m^{\left(j\right)}\right)}^{T}$$

We use the trace of the dispersion matrix as a measure of the sample divergence. That is $tr(S_{W}^{\left(i\right)})$ represents the divergence of each sample in $i^{th}$ class to the mean vector. And the $tr(S_{W})$ is the mean measure of the feature variance of all the classes. The $S_{B}^{\left(ij\right)}$ donates the dispersion between $i^{th}$ and $j^{th}$ classes while the $S_{B}$ is a measurement of the mean dispersion between the mean of each class and the global mean vector. So the feature extracted by the SDAE should make the inner-class divergence as small as possible and the outer-class divergence as large as possible. 

We selected six types of sample data and compared the sample dispersion before and after using SDAE. Table 2 lists the total inner and outer class divergence of the Original data and the extracted feature

The inner-class divergence has been reduced by nearly 97.5% from the original data to the extracted feature. Although the outer-class divergence also decreases, the proportion of the decline is much smaller than the inter-class divergence. For the original data, the inner-class divergence is greater than the outer-class divergence. But for the extracted features, the outer-class divergence is more than four times the inner-class divergence.

Table 3 and Table 4 list the specific inner and outer class divergence of the Original data and the Extracted feature. The upper left corner is the calculation result of the original data, and the lower right corner is for the extracted features. As can be seen from the table comparison, the feature extracted by SDAE has a significant effect. For example, the inner-class divergence of WALK is 10.50, larger than the outer-class divergence between WALK and SIT on the original data. After the feature extraction, the inner-class divergence changes to 0.09 while the outer-class divergence becomes 0.49, which is more than five times that. Therefore, both total and specific divergence demonstrate that the SDAE has obvious advantages in excavating the hidden features of various types of data.

**Figure 2:**
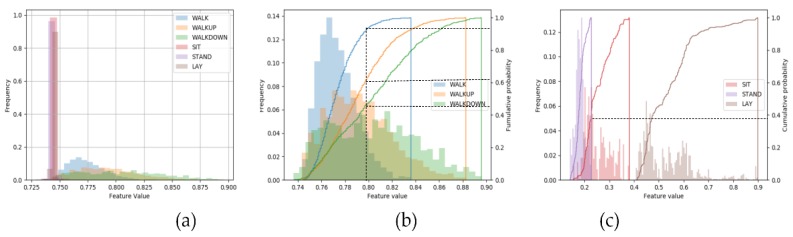
The feature values between different categories. (**a**) Shows the different distributions of a certain feature value between static classes and dynamic classes. (**b**) Displays the data distribution and cumulative probability of a certain feature on the dynamic class and (**c**) shows that on the static class.

To visually verify the validity of the extracted features from the SDAE model, we selected several features and made the numerical distribution diagram of all the samples in each category on a certain feature, which is shown in Figure 2. 

Figure 2a show that the feature values of the three categories in the static state are 100% distributed in the range of 0.735−0.750, while the values of the other three categories in the dynamic state are mostly distributed in the range of 0.75−0.90, with almost no overlap. In Figure 2b, although the distribution of the sixth feature value among the three dynamic categories overlaps to a certain extent, it still has strong classification ability. For example, 93% values of the WALK class are distributed between 0.74 and 0.80, while 61% for WALKUP class and for WALKDOWN, only 43%. As for the sixty-ninth feature on the three static classes shown in Figure 2c, the classification effect is particularly noticeable. The feature values of the LAY class are distributed between 0.4 and 0.9, with no overlap with other classes. For the STAND and SIT, there is only 38% overlap.

Although a single feature has shown a certain classification ability, it still has a lot of limitations. Therefore, we need a powerful classifier to deal with the 90-dimensional features learned by SDAE to achieve the best classification effect.

### 2.4. Supervised Classification

To make full use of the features extracted by SDAE for high-precision classification, we selected the LGB algorithm as a supervised classification method. This section gives a simple introduction to the advantages and calculation methods of LGB, and explains the Boosting K-fold algorithm proposed in this paper.

#### 2.4.1. Classification Algorithm

With the labeled training dataset $\mathbb{C}{=\{(e}_{{\vec{x}}_{i}},y_{i}{)\}}_{i=1}^{N}$ gained from the unsupervised feature extraction layer, the LGB algorithm will be used. LGB is a new GBDT implementation with Gradient-based One-Side Sampling (GOSS) and Exclusive Feature Bundling (EFB) that meet the requirements of efficiency and scalability under the situation of high dimension and a large amount of data. Researches show that LGB will speed up the training process of conventional GBDT by up to 20 times while achieving almost the same accuracy [48].

#### 2.4.2. Boosting K-fold

In k-fold cross-validation, the original sample is randomly partitioned into k equal sized subsamples. Of the k subsamples, a single subsample is retained as the validation data for testing the model, and the remaining k − 1 subsamples are used as training data. The cross-validation process is then repeated k times, with each of the k subsamples used exactly once as the validation data. 

**Figure 3:**
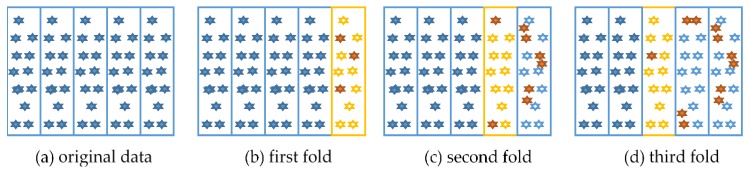
The schematic diagram of boosting K-Fold LGB. The training sets of each fold are determined by the previous fold’s predicted results.

In this paper, we use the idea of boosting into the process of five-fold cross-validation. The change process of the dataset is shown in Figure 3. In this algorithm, the five-fold cross-validation is a serial process. At first, the original data, as shown in Figure 3a, was initially divided into five parts. During the first fold training, the samples that were misclassified in the verification set were selected Figure 3b. In the second fold of training, the misclassified samples we first copied to achieve the purpose of increasing the weight, and then were used as the training set Figure 3c. So on, the error samples in the verification set were marked and copied for the third fold of training Figure 3d. Repeat this process until all training is completed.

The pseudo code of Algorithm 2 shows the implementation of Boosting K-Fold LGB. This algorithm receives the whole train data and label named “X” and “Y” as input. The trained LGB models will be used for prediction and this process is not shown in Algorithm 2.

**Algorithm 2:** Supervised Feature Classification**Input:** Training data named X, training label named Y, test data named X_test **Output:** The predict result of X_test 1. kfold_data = StratifiedKFold (folds_num = 5) 2. misjudgedX = [] 3. misjudgedY = [] 4. **for** train_index, val_index in kfold_data.split(X, Y) 5.  tra_x, val_x, tra_y, eval_y = X[train_index], X[val_index], Y[train_index], Y[val_index] 6.  **if** len(misjudgedX) ! = 0 7.   tra_x = vstack ([tra_x, misjudgedX]) 8.   tra_y = hstack([tra_y, misjudgedY]) 9.  **end if** 10.  model = lightgbm.train(tra_x, tra_y) 11.  val_pred = model.predict(val_x) 12.  misjudgedX = val_x[argwhere(val_y ! = val_pred)] 13.  misjudgedY = val_y[argwhere(val_y ! = val_pred)] 14.  test_pred.append(model.predict(X_test)) 15. **end for** 16. final_pred = [] 17. **for** line in test_pred 18.  final_pred.append(argmax(bincount(line))) 19. **end for** 20. return final_pred

### 2.5. Models for Comparison

In this section, we provide a brief introduction to four algorithms that are used in the literature for comparison with the method proposed in this paper. These are single SDAE, XGB, CNN, and CNN + Statistic Features.

#### 2.5.1. HAR Based on SingleSDAE

**Figure 4:**
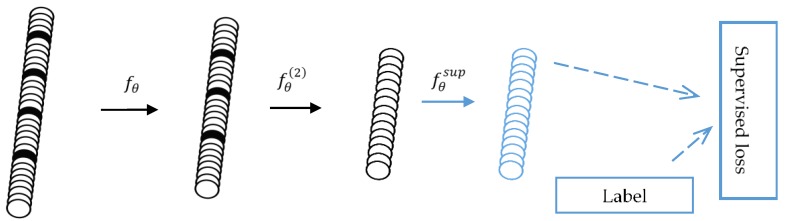
The single SDAE network for classification. After the training of stacking denoising encoders, an output layer is added on the top of the encoding network. By performing the gradient descent on the supervised loss, the classification result of single SDAE can be obtained.

A single SDAE model can also be directly used for multiple classification problems. In this paper, the effects of single SDAE and SDAE+LGB were also compared. The pre-train phase of single SDAE is the same as 3-B. And the difference between them is that a softmax layer will be superimposed on the trained encoding network for category prediction for single SDAE. The reverse propagation process is the same as that of a neural network. The algorithm schematic diagram is shown in Figure 4.

#### 2.5.2. HAR Based on XGB

The XGB algorithm is one of the common machine learning algorithms in HAR. In this paper, we construct a complete set of feature engineering by studying the internal laws of data sets. Then the XGB algorithm is called for classification. The accuracy of XGB will be compared with the algorithm proposed in this paper. The characteristics of the data are constructed as Table 5.

#### 2.5.3. HAR Based on CNN

**Figure 5:**
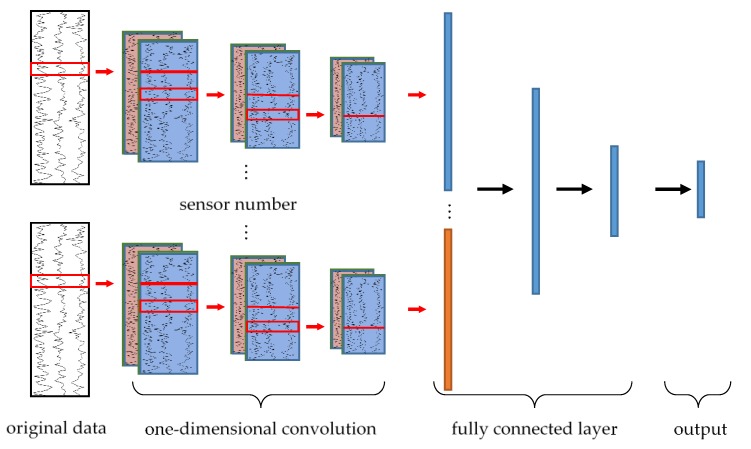
The convolutional neural network (CNN) network of human activity recognition. Each convolutional network is for a set of data in a sensor. Then the outputs of the convolutional network are spliced together to be the input of the fully connected layer.

As a classic supervised deep learning method, CNN can also be used in HAR. This paper studied the accuracy under the CNN algorithm either. Taking the UCI-HAR data set as an example, the CNN network structure adopted in this paper is shown in Figure 5. One dimensional convolution operation was performed on the three sensor data respectively. The characteristics obtained by convolution were stretched into a one-dimensional vector. Then the completed input vector of the fully connected layer can be obtained by splicing the feature vector of each sensor. After three full connection operations, the output will be obtained, in which the $i^{th}$-dimension represents the probability that the current sample belongs to the $i^{th}$-class. 

Considering that CNN is only an algorithm for comparison, we directly adopted the optimal parameters in the paper [27], only slightly changing the network structure of CNN according to different data formats of the input.

#### 2.5.4. HAR Based on CNN+ Statistic Features

We also implement a state-of-art HAR algorithm for comparison with our own method. The method proposed in [13] presents a user-independent deep learning-based approach for online human activity recognition by using CNN for local feature extraction together with simple statistical features that preserve information about the global form of time series. The results published in [13] show that this method demonstrates state-of-the-art performance while requiring low computational cost and no manual feature engineering. 

## 3. Experiments and Evaluation

To evaluate the performance of the proposed algorithm, we carried out a set of experiments described in this section.

### 3.1. Datasets

For the evaluation of the generalization ability of the algorithm we proposed, we tested four datasets from three typical scenarios. These datasets are elaborated below and the number of specific sample for each category of each dataset is shown in Table 6.

**The Human Moving Modes with Pressure (HMMwithPre)**: This dataset is from a variety of smartphones (HUAWEI NXT-TL00, NXT-AL10, Samsung G9200, MIX 2, and MI 5s) positioned horizontally in the user’s hand to collect data from an accelerometer, gyroscope, magnetic, and air pressure sensor at a 100 Hz sampling rate. Twenty-five (25) subjects participated in data collection: 20 men and 5 women from 20 to 50 years old, of 165−192 cm height and 48−80 kg weight. Let n represents the length of the sequence. The processed data were built from 50%-overlapping sliding windows with 256 samples. Since the sampling frequency was 100 Hz, each data frame lasted 2.56 s, with every new frame available every 1.28 s. Finally, the sample shape obtained is ((n|128)−1,256,10).

**The Human Moving Modes without Pressure (HMMwithoutPre)**: This dataset is a variation of the HMMwithPre, which was derived from the original HMMwithPre data by removing the pressure sensor data. With the same sliding window size and step as HMMwithPre, the final sample shape is ((n|128)−1,256,9).

**The Human Static Behavior Dataset (HSBD)**: This dataset (https://archive.ics.uci.edu/ml/ datasets/Human+Activity+Recognition+Using+Smartphones) is from a single smartphone (Samsung Galaxy S2) positioned on the user’s waist to collect the total accelerometer, the estimated body accelerometer and gyroscope data at a 50 Hz sampling rate. Thirty (30) subjects aged 19−48 years participated in data collection. The processed data were built from no-overlapping sliding windows with 2.56 s. Since the sampling frequency was 50 Hz, each data frame contains 128 samples. Finally, the sample shape obtained is (n|128,128,9)

**The Human Dynamic Behavior Dataset (HDBD)**: This dataset (http://archive.ics.uci.edu/ml/ datasets/Smartphone-Based+Recognition+of+Human+Activities+and+Postural+Transitions) is from a single smartphone (Samsung Galaxy S2) positioned on the user’s waist to collect the total accelerometer, the estimated body accelerometer and gyroscope data at a 50 Hz sampling rate. It is an updated version of the HSBD. After removing the data which has the same label with HSBD, the training samples were extracted. Considering the small number of datasets, a 0.16 s sliding window is adapted to obtain the samples, the shape of which is ((n|8)−1,128,9).

### 3.2. Evaluation Metrics

In order to comprehensively evaluate the performance of HAR, we used four evaluation *accuracy (A), precision (P), recall (R), F1-score (F1)* to evaluate the classification results. For this multi-classification problem, the calculation steps of P, R, and F1 is shown below.

Step1: For each activity category, count the number of samples of predicting this class as this class (TP), predicting other classes as this class (FP), predicting this class as other classes (TN).

Step2: Calculate $P_{k},R_{k},F1_{k}$ under each category by the statistics of the first step. The calculation formula is as follows:

(11) $$P_{k}=\frac{TP}{TP+FP},R_{k}=\frac{TP}{TP+FN},f1_{k}=\frac{2\times P_{k}\times R_{k}}{P_{k}+R_{k}}$$

Step3: Average the results under all the categories obtained in the second step.

### 3.3. Network Structure of SDAE

SDAE network is composed of multiple encoding layers and decoding layers, the number of cells of which are different. The network structure of our SDAE for each dataset is summarized in Table 7, where the n_layer represents the number of the encoder layers, the n_hidden represents the number of cells in each layer of the encoding layer, and the dropout represents the rate at which input data is discarded.

### 3.4. Classification Performance

**Figure 6:**
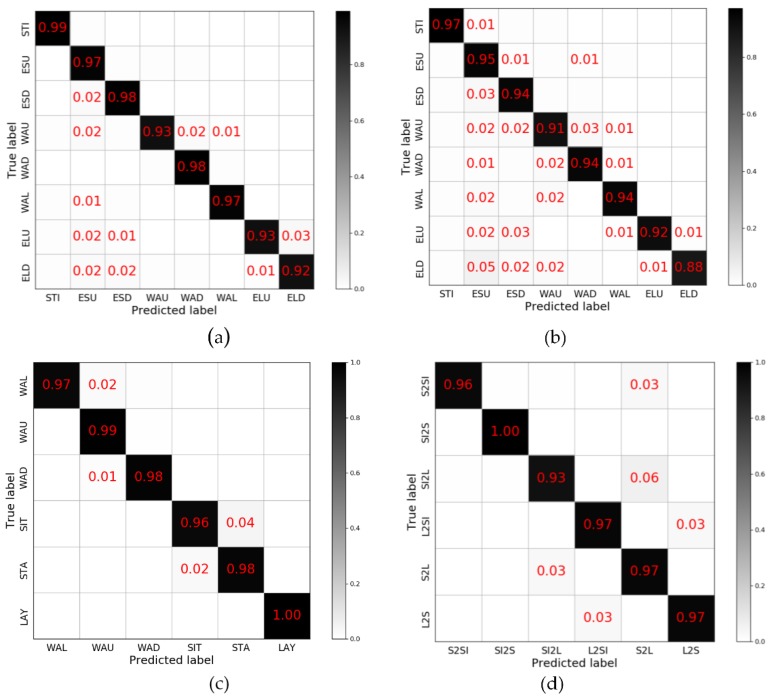
The confusion matrix of SDAE+LGB on each dataset: (**a**) HMMwithPre, (**b**) HMMwithoutPre, (**c**) HSBD, (**d**) HDBD. The Number in each cell is the probability of the current predicted result, while blank represents the probability is below 0.01.

In the first experiment, we evaluated our proposed method on the HMMwithPre, HMMwithoutPre, HSBD, and HDBD datasets. The recognition results are presented as confusion matrices in Figure 6 and summarized as average recognition accuracy in Table 8.

For each dataset, the data on the diagonal occupies an absolute proportion. With the HMMwithPre dataset, the recognition accuracy is 95.73% while there is a large possibility that the elevator_up and elevator_down are misjudged and also 1% of the walking_up and walking_down data are judged as walking. As for HMMwithoutPre dataset, all but the stilling categories have a certain probability of being misjudged. Among them, the probability that the elevator_down is judged as others and the others being judged as escalator up is the highest. For HSBD, three categories of motion (walking, walking up, walking_down) and three categories of rest (sitting, lying, standing) can be perfectly separated. In the rest categories, the misjudgment rate between standing and sitting was higher. With the HDBD, the samples of misjudgement mainly focus on the discrimination of standing or sitting to lying, and lying to standing or sitting, which is consistent with the classification result of HSBD.

The experiment result has shown that the algorithm proposed in this paper achieves a good classification effect on multiple datasets, especially on the discrimination of motion and rest data. In the categories of human moving modes, escalator and elevator have a relatively high misjudgement rate, while for the data of user behavior, the distinction between standing and sitting is the difficulty of the classification.

### 3.5. Comparison of Different Models

In the second experiment, to compare the other classification performance with the algorithm proposed in this paper, we implemented additional single SDAE algorithms, XGB, CNN, and the CNN + Statistical features algorithm proposed in [13]. The experimental results are shown below.

#### 3.5.1. Comparison with Single SDAE

The classification result of single SDAE is shown in Table 9 comparing with SDAE+LGB. The difference between them is that the former uses a fully connected layer for classifying rather than LGB. As shown in the table, the accuracy of single SDAE is about 10% lower than that of SDAE+LGB but varies little among the four datasets, which verifies the robustness of SDAE to extract effective features.

#### 3.5.2. Comparison with XGB

Table 10 shows the evaluation score of XGB. As for HMMwithPre, by the XGB algorithm achieves an accuracy of 95.06% which fully demonstrates the effectiveness of the proposed feature. However, the 10s’ sliding window of pressure data will cause a long time delay and affect the real-time performance of recognition. With the HMMwithoutPre dataset, the performance XGB dropped significantly by almost 10% simply because it was missing four pressure-dependent features. Experimental results show that the performance of XGB greatly depends on the effectiveness of features.

#### 3.5.3. Comparison with CNN

The performance of CNN is shown in Table 11 with an average accuracy of 89.52% on the four datasets. The experimental results show that the CNN algorithm has good robustness and will get similar results on multiple data sets. However, CNN still has shortcomings in feature extraction, which limits its accuracy.

#### 3.5.4. Comparison with CNN+Statistical Features

The comparison results on the all datasets are shown in**Table 12**. As can be seen from the table, the accuracy of the CNN + Statistical features algorithm varies greatly on distinct datasets, from 78.11% to 97.63%. Compared with CNN, this algorithm has a great improvement in HSBD, but a sharp decrease in HDBD. This indicates that the algorithm is not robust, mainly because the features extracted manually are not universal.

#### 3.5.5. Analysis of Comparison Results

From the experiment results, the highest accuracy on different classification methods is achieved by using SDAE+LGB. As for the single SDAE algorithm, the learning ability of the fully connected layer added after the encoding layers are limited. It’s difficult for it to make full use of the features acquired by encoding–decoding network learning. For the XGB algorithm, the accuracy of recognition largely depends on the effectiveness of the extracted feature. Meanwhile the accuracy of the CNN algorithm is affected by many super parameters, and it’s difficult to find an optimal combination to achieve the ideal accuracy of identification network. When together with the statistical features, the robustness of CNN will drop significantly.

## 4. Conclusions

In this paper, we propose a human activity recognition algorithm combining the feature extraction ability of SDAE and the classification ability of LGB and demonstrate its capability to produce a robust HAR. The evaluation was performed on four distinct datasets under different combination of sensors, various sensor positions, and three typical application scenarios which are human moving modes, current static behavior, and dynamic behavior change. For comparison, we also implemented the single SDAE, XGB, CNN, and a state-of-art algorithm and compared it with the SDAE+LGB algorithm on each dataset. 

Extensive experimental results demonstrate that our proposed algorithm is more generic and robust than other state-of-art algorithms. There are two main reasons for this. One is that the features learned by SDAE are more capable of showing the variation law of sensor data than those constructed manually. The other is that for the same features, the classification capability of LGB is better than that of a simple fully connected layer.

For the future work, we plan to conduct further research along the following lines. First, we will explore the usage of unlabeled data generated during the user’s use to improve the existing model incrementally. Second, we will construct an effective indoor positioning algorithm by combining the classification results of human moving modes with Pedestrian Dead Reckoning (PDR). Third, we will translate the classification results of HAR into practical semantic layer expression, which can provide suggestions for human daily life.

## 5. Patents

The proposed method is applying for a patent and now has been handed over to the agency.

## Figures and Tables

**Table 1:** The further experimental results of the related work.

Reference	Dataset	Algorithm	Accuracy
[37] [37] [25] [26] [20] [15] [27] [13] [17] [28] [29] [36] [19] [38] [39]	WISDM UCI UCI Non-public Non-public Non-public Non-public UCI RealWorld HAR Non-public PAMAP2 Non-public HASC UCI UCI	Artificial features + Dropout Artificial features + Random Forest Artificial features + SVM Artificial features + SVM Hierarchical artificial features + LR//DT/SVM/XGB Artificial features + MP+LigitBoost+SVM CNN CNN + statistical features CNN + statistical features binary sensor convolution neutral network deep dilated convolution + long short term memory deep neural networks + hidden Markov models deep recurrent neural network sparse autoencoder + SVM stacked autoencoder	85.36% 76.26% 89.00% 85.59% - 91.15% 93.80% 97.63% 98.00% 94.70% - 93.52% 95.42% 92.16% 97.90%

**Table 2:** The total inner and outer class divergence of the Original data and the Extracted feature.

Evaluation Indicator	Original Data	Extracted Feature
inner-class divergence	8.0264	0.2064
outer-class divergence	6.9440	0.8646

**Table 3:** The specific inner and outer class divergence of the Original data.

Category	WALK	WALKUP	WALKDOWN	SIT	STAND	LAY
WALK	10.50	0.48	0.06	3.95	0.05	45.21
WALKUP	13.25	0.77	6.79	0.77	50.39	0.05
WALKDOWN	17.73	3.30	0.05	43.29	0.77	3.95
SIT	2.46	3.29	26.81	0.05	6.79	0.06
STAND	0.98	43.56	3.29	3.30	0.77	0.48
LAY	7.03	0.98	2.46	17.73	13.25	10.50

**Table 4:** The specific inner and outer class divergence of the Extracted feature.

Category	WALK	WALKUP	WALKDOWN	SIT	STAND	LAY
WALK	0.09	0.23	0.32	0.49	0.12	5.69
WALKUP	0.23	0.21	0.4	0.67	0.64	6.03
WALKDOWN	0.32	0.4	0.23	0.49	0.39	5.63
SIT	0.49	0.67	0.49	0.37	0.41	3.38
STAND	0.12	0.64	0.39	0.41	0.04	5.48
LAY	5.69	6.03	5.63	3.38	5.48	0.27

**Table 5:** The feature engineering of XGB algorithm.

Sensors	Cluster	Features
Acceleration	Vertical component	mean, variance, standard deviation, median, minimum, maximum, range, quartile
	Horizontal component	mean, variance, standard deviation, median, minimum, maximum, range, quartile
Acceleration &Gyroscope & Magnetic	Modulus value	mean, variance, standard deviation, median, minimum, maximum, range, quartile, kurtosis, skewness, root mean, square, integral, double integral, autocorrelation, 7 FFT features
	Three axes value	Pearson correlation coefficient between three axes
Pressure	6s’s windows	Change value, standard deviation
	10s’s windows	Change value, standard deviation

**Table 6:** The sample number of each category on each dataset. The HMM contains both HMMwithPre and HMMwithoutPre.

Dataset	Category	Abbreviation	Number of Samples	Sample Percent of Each Human Activity
HMM	Stilling Walking Elevator_up Elevator_down Escalator_up Escalator_down Walking_up Walking_down	STI WAL ELU ELD ESU ESD WAU WAD	1325 2216 685 730 2216 1598 864 1029	12.42% 20.78% 6.42% 6.84% 20.78% 14.98% 8.10% 9.65%
HSBD	Walking Walking_up Walking_down Standing Sitting Laying	WAL WAU WAD STA SIT LAY	1772 1544 1406 1777 1906 1944	17.12% 14.91% 13.58% 17.17% 18.41% 18.78%
HDBD	Stand-to-sit Sit-to-stand Sit-to-lie Lie-to-sit Stand-to-lie Lie-to-stand	S2SI SI2S SI2L L2SI S2L L2S	697 192 1193 879 1753 870	12.48% 3.43% 21.36% 15.74% 31.39% 15.58%

**Table 7:** The network structure for SDAE.

Dataset	n_Layer	n_Hidden	Dropout	Batchsize	Epoch
HMMwithPre	3	[150,70,20] for Pre others [400,200,40]	0.4	32	20
HMMwithoutPre	3	[400,200,30]	0.4	32	4
HSBD	2	[100,30]	0.4	32	2
HDBD	1	[30]	0.4	32	20

**Table 8:** Average recognition accuracy of our proposed method on the different datasets.

Dataset	Accuracy
HMMwithPre HMMwithoutPre HSBD HDBD	95.73% 93.70% 98.22% 96.31%

**Table 9:** The evaluation score of single SDAE on four datasets.

Dataset	Model	Accuracy	Precision	Recall	F1-score
HMMwithPre	SDAE SDAE+LGB	86.04% 95.73%	87.22% 95.76%	86.04% 95.73%	86.01% 95.73%
HMMwithoutPre	SDAE SDAE+LGB	84.42% 93.70%	84.77% 93.74%	84.42% 93.70%	84.37% 93.70%
HSBD	SDAE SDAE+LGB	84.63% 98.22%	86.58% 98.23%	84.62% 98.22%	84.79% 98.22%
HDBD	SDAE SDAE+LGB	79.14% 96.31%	80.65% 96.33%	79.14% 96.31%	78.38% 96.31%

**Table 10:** The evaluation score of XGB on four datasets.

Dataset	Model	Accuracy	Precision	Recall	F1-score
HMMwithPre	XGB SDAE+LGB	95.06% 95.73%	95.06% 95.76%	95.06% 95.73%	95.03% 95.73%
HMMwithoutPre	XGB SDAE+LGB	85.47% 93.70%	85.41% 93.74%	85.47% 93.70%	85.12% 93.70%
HSBD	XGB SDAE+LGB	94.05% 98.22%	94.07% 98.23%	94.05% 98.22%	94.05% 98.22%
HDBD	XGB SDAE+LGB	80.12% 96.31%	80.21% 96.33%	80.12% 96.31%	79.99% 96.31%

**Table 11:** The evaluation score of CNN on four datasets.

Dataset	Model	Accuracy	Precision	Recall	F1-score
HMMwithPre	CNN SDAE+LGB	90.77% 95.73%	90.77% 95.76%	90.57% 95.73%	90.77% 95.73%
HMMwithoutPre	CNN SDAE+LGB	88.64% 93.70%	88.31% 93.74%	88.42% 93.70%	88.31% 93.70%
HSBD	CNN SDAE+LGB	85.83% 98.22%	85.84% 98.23%	85.83% 98.22%	85.83% 98.22%
HDBD	CNN SDAE+LGB	92.84% 96.31%	93.84% 96.33%	92.97% 96.31%	92.97% 96.31%

**Table 12:** The comparison of evaluation score of [13] on four datasets.

Dataset	Model	Accuracy	Precision	Recall	F1-score
HMMwithPre	[13] SDAE+LGB	93.24% 95.73%	93.15% 95.76%	93.24% 95.73%	93.19% 95.73%
HMMwithoutPre	[13] SDAE+LGB	86.31% 93.70%	86.15% 93.74%	86.31% 93.70%	86.05% 93.70%
HSBD	[13] SDAE+LGB	97.63% 98.22%	97.68% 98.23%	97.63% 98.22%	97.62% 98.22%
HDBD	[13] SDAE+LGB	78.11% 96.31%	78.11% 96.33%	78.11% 96.31%	78.10% 96.31%
